# Influence of metabolic syndrome on the relationship between fatty acids and the selected parameters in men with benign prostatic hyperplasia

**DOI:** 10.18632/aging.101850

**Published:** 2019-03-13

**Authors:** Katarzyna Grzesiak, Aleksandra Rył, Weronika Ratajczak, Ewa Stachowska, Iwona Rotter, Marcin Słojewski, Olimpia Sipak, Kinga Walczakiewicz, Maria Laszczyńska

**Affiliations:** 1 Department of Histology and Developmental Biology, Pomeranian Medical University, 71-210 Szczecin, Poland; 2 Department of Medical Rehabilitation and Clinical Physiotherapy, Pomeranian Medical University, 71-210 Szczecin, Poland; 3 Department of Biochemistry and Human Nutrition, Pomeranian Medical University, 71-460 Szczecin, Poland; 4 Department of Urology and Urological Oncology, Pomeranian Medical University, 70-111 Szczecin, Poland; 5 Department of Obstetrics and Pathology of Pregnancy, Pomeranian Medical University, 71-210 Szczecin, Poland

**Keywords:** benign prostatic hyperplasia, fatty acids, metabolic syndrome

## Abstract

The purpose of our investigation was to analyze the relationship between the serum levels of fatty acids and their metabolites and the levels of the selected metabolic and hormonal parameters in patients with benign prostatic hyperplasia (BPH) with regard to concomitant metabolic syndrome (MetS). We determined serum concentrations of total (TT) and free testosterone (FT), insulin (I), dehydroepiandrosterone sulphate (DHEAS), luteinizing hormone and insulin-like growth factor 1 (IGF-1) and sex hormone-binding globulin (SHBG). Gas chromatography was performed. The patients differed in terms of hormone levels, but only the differences in SHBG and IGF-1 levels were statistically significant. Analysis of the levels of polysaturated fatty acids in BPH patients showed that MetS contributed to changes in the levels of these acids. We also analyzed the relationship between the levels of fatty acids and diagnostic parameters for MetS. Particular abnormalities were associated with single changes in the levels of fatty acids. In the diabetic patients, changes in the levels of pentadecanoic acid, heptadecanoic acid and cis-11-eicosenoic acid were demonstrated. Our findings indicate the necessity for further investigation concerning the levels of fatty acids and their impact on the development of MetS, as well as the course and clinical picture of BPH.

## Introduction

Benign prostatic hyperplasia (BPH) belongs to the most common urological diseases afflicting men over 50 years of age [1]. Its development is underlain by metabolic disorders [2] and an inflammatory process in the prostate gland [3,4]. Numerous epidemiological studies have emphasized the link between metabolic syndrome (MetS) and prostatic enlargement that is secondary to BPH [2,5,6]. MetS can be regarded as chronic inflammation of the body, which involves tissue remodeling in such health problems as atherosclerosis, diabetes, hypertension, and aging-related processes [7]. Since interleukin 8 (IL-8) is secreted in the body’s response to changes in the level of oxidation of low density lipoprotein (LDL) and changes in insulin levels, decreased levels of high density lipoprotein (HDL) and increased levels of triglycerides (TG), observed in MetS, are significantly associated with inflammation of the prostate gland [8]. These factors may suggest the connection between the processes underlying the development of MetS and BPH-related inflammation [9,10].

**Figure 1:**
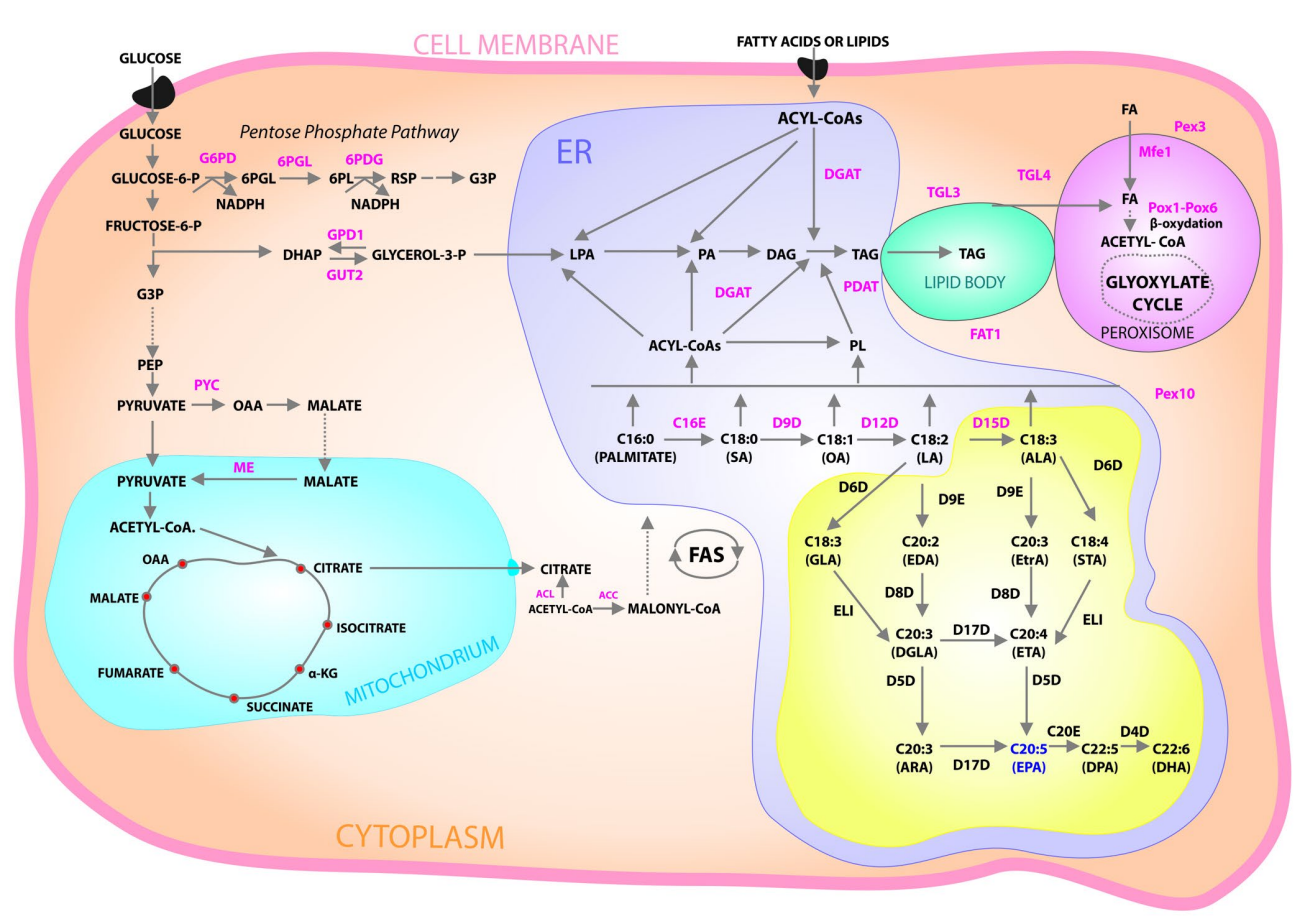
**Cytosolic glucose enters glycolysis and pentose phosphate pathways.** Pyruvate enters mitochondria where it is converted into acetyl-CoA then used in TCA cycle. Excess citrate is transported from the mitochondria into cytosol. ATP citrate lyase (ACL) converts the cytosolic citrate into acetyl-CoA that is converted into malonyl-CoA by acetyl-CoA carboxylase (ACC), the first committing step of fatty acid (FA) synthesis. After FA synthesis, triacylglycerol (TAG) is synthesized in the endoplasmic reticulum (ER) via Kennedy pathway and then accumulated in lipid bodies (LB). Acyl-CoA is used for acylation of glycerol-3- phosphate to form lysophosphatidic acid (LPA) that is further acylated to form phosphatidicacid (PA). PA is dephosphorylated to form diacylglycerol (DAG), which is then acylated to produce TAG catalyzed by DAG acyltransferase (DGAT). TAG can also be synthesized by phospholipid (PL):DAG acyltransferase (PDAT) using PL and DAG as substrates. Ex novo FA accumulation also uses Kennedy pathway. FA is metabolized by β- oxidation pathway in peroxisome. Abbreviations: 6PGL, 6-Phosphogluconolactonase; 6PDG, 6-Phosphogluconate dehydrogenase; α-KG, alpha-ketoglutarate; DHAP, dihydroxyacetone phosphate; FAT1, FAT Atypical Cadherin 1; G3P, glyceraldehyde3-phosphate; G6PD, glucose-6-phosphate dehydrogenase; GPD1, glycerol-3-phosphate dehydrogenase; GUT2, glycerol-kinase; Mfe1, multifunctional enzyme 1; ME, malic enzyme; OAA, oxaloacetate; PEP, phosphoenolpyruvate; Pex3 and Pex10, peroxisome biogenesis factor 3 and 10, respectively; Pox1 to Pox6, acyl-CoA oxidases 1–6, respectively; TGL3 and TGL4, TAG lipase 3 and 4, respectively; PYC, pyruvatecarboxylase; TCA, tricarboxylic acid cycle. Yellow box: schematic diagram of aerobic pathways for ω-3 and ω-6 FA biosynthesis. Abbreviations: C16E, EL1, C20E and D9E are C16/C18, C18, C20/C22, Δ-9 elongases, respectively. D4D, D5D, D6D, D8D, D9D, D12D, D15D and D17D are Δ-4, Δ-5, Δ-6, Δ-8, Δ-9, Δ-12, Δ-15, and Δ-17 desaturases, respectively.

Fatty acids (FA) play an important role in the human innate immune system. Chemically classified as fatty acids with one double bond, monounsaturated fatty acids (MUFA) are considered as potentially reducing the risk of MetS [11]. Polyunsaturated fatty acids (PUFA) with two or more double bonds are essential for the proper functioning of the body (including the cardiovascular system), and for generating the body's inflammatory response [12,13]. The main mediators of inflammation include arachidonic acid (AA) and linolenic acid (LA) [14,15], as well as their oxidation products—prostaglandins (PGs), thromboxanes (TXs), hydroxyeicosatetraenoic acids (HETEs), and hydroxyoctadecadienoic acids (HODEs)—which also underlie the pathogenesis of atherosclerosis [16,17], Figure 1. Interest in the metabolites of polyunsaturated fatty acids, produced by the lipoxygenase (LOX) pathway, has visibly grown in recent years. Particular attention is paid to resolvins derived from eicosapentaenoic acid (EPA) and docosahexaenoic acid (DHA). Resolvins are lipid mediators of the inflammatory process in the body. Their biosynthesis is significantly higher in tissues affected by inflammation, and during such processes as modulation of the immune response, hormone secretion, angiogenesis, as well as cell growth, proliferation, and adhesion (playing a key role in the development of cancerous changes) [18]. No studies have been conducted so far that assess the connection between fatty acids and particular MetS parameters in patients with BPH.

The purpose of our investigation was to analyze the relationship between the serum levels of fatty acids and their metabolites and the levels of the selected metabolic and hormonal parameters in patients with BPH with regard to concomitant MetS.

## RESULTS

Our study demonstrated statistically significant differences in the levels of the diagnostic parameters for MetS (waist circumference, TG, HDL, FPG) between the group with MetS and the group without this syndrome (p < 0.001) (Table 1). The patients differed in terms of hormone levels, but only the differences in the levels of SHBG (p = 0.016) and insulin-like growth factor 1 (IGF-1) (p = 0.017) were statistically significant. Analysis of the levels of polysaturated fatty acids in the BPH patients (both with and without MetS) showed that MetS contributed to changes in the levels of the following acids: C10:0 capric acid, C14:0 myristic acid, C14:1 myristoleic acid, C15:0 pentadecanoic acid, C16:0 palmitic acid, C16:1 palmitoleic acid, C17:0 heptadecanoic acid, C18:1n9 cis/trans oleic acid, C18:1 transvaccenic acid, C18:3n3 linolenic acid, and C22:1 cis-11-eicosenoic acid. The ratio of C18:3n6 gamma-linolenic acid to C18:2n6c linoleic acid was 0.01, while the ratio of C18:3n3 linolenic acid to C18:2n6c linoleic acid was 0.05.

**Table 1:** Relationships between the studied parameters in BPH patients with and without MetS.

**Variables**	**Patients with BPH and without MetS** **n=101**				**Patients with BPH and with MetS** **n=53**				**p**
	**X**	**SD**	**Min**	**Maks**	**X**	**SD**	**Min**	**Maks**	
**Anthropometric parameters**									
**Age [years]**	67.15	7.10	46.00	80.00	66.62	7.22	44.00	90.00	0.551
**Body weight [kg]**	79.43	12.80	54.00	125.00	93.85	16.29	58.00	136.00	<0.001*
**BMI [kg/m2]**	27.28	6.01	19.44	71.62	31.36	5.30	22.10	48.08	<0.001*
**WC [cm]**	94.85	9.94	70.00	122.00	110.47	10.97	93.00	138.00	<0.001*
**Biochemical parameters in serum**									
**TG [mg/dl]**	117.31	47.93	46.00	303.00	169.51	89.06	76.00	625.00	<0.001*
**TCh [mg/dl]**	198.98	44.18	102.00	322.00	194.89	49.85	109.00	361.00	0.473
**HDL [mg/dl]**	73.18	16.12	26.00	129.00	66.72	11.23	47.00	100.00	0.009*
**LDL [mg/dl]**	102.67	41.31	21.00	203.50	92.68	41.39	18.00	212.10	0.155
**FPG [mg/dl]**	90.47	21.39	47.60	161.00	97.20	37.03	26.40	156.00	0.001*
**Hormonal parameters and proteins in serum**									
**HOMA**	3.05	3.29	0.01	18.81	4.12	5.03	0.02	21.72	0.488
**DHEA [ug/mg]**	0.56	0.92	0.00	6.57	0.62	1.14	0.00	7.89	0.941
**E2 [ pg/ml]**	49.32	24.48	11.89	123.56	46.87	29.79	11.00	176.03	0.290
**SHGB nmol/mg]**	45.98	17.16	11.90	102.68	40.58	17.22	8.19	107.30	0.016*
**LH [mIU/ml]**	8.65	6.06	0.90	38.53	7.26	4.86	1.11	27.18	0.181
**TT [ng/ml]**	4.38	1.99	0.71	10.15	4.15	2.12	1.23	11.13	0.280
**TF [pg/ml]**	7.85	6.22	0.20	34.83	6.82	7.58	0.08	48.62	0.146
**IGF-1 [ng/ml]**	82.85	39.31	33.81	361.90	91.27	28.84	39.07	169.90	0.017*
**I [µIU/ml]**	15.03	11.82	0.10	59.55	17.06	14.86	0.00	62.88	0.589
**Fatty acids in serum**									
**C10:0 Capric acid [mg/ml]**	0.03	0.01	0.01	0.06	0.03	0.01	0.01	0.07	0.021*
**C12:0 Lauric acid [mg/ml]**	0.01	0.00	0.00	0.03	0.01	0.00	0.00	0.02	0.282
**C14:0 Myristic acid [mg/ml]**	0.06	0.02	0.02	0.12	0.08	0.04	0.03	0.20	0.001*
**C14:1 Myristolenic acid [mg/ml]**	0.01	0.00	0.01	0.02	0.01	0.00	0.01	0.03	0.026*
**C15:0 Pentadecanoid acid [mg/ml]**	0.01	0.00	0.01	0.03	0.02	0.01	0.01	0.03	0.003*
**C15:1 cis-10-pentadecanoid acid [mg/ml]**	0.00	0.00	0.00	0.01	0.00	0.00	0.00	0.01	0.629
**C16:0 Palmitic acid [mg/ml]**	1.88	0.44	1.07	2.91	2.17	0.69	1.27	4.31	0.021*
**C16:1 Palmitoleic acid [mg/ml]**	0.12	0.05	0.04	0.27	0.16	0.08	0.06	0.47	0.002*
**C17:0 Heptadecanoid acid [mg/ml]**	0.02	0.01	0.01	0.04	0.02	0.01	0.01	0.04	0.004*
**C17:1 cis-10- Heptadecanoid acid [mg/ml]**	0.00	0.00	0.00	0.01	0.00	0.00	0.00	0.01	0.193
**C18:0 Stearic acid [mg/ml]**	0.69	0.13	0.45	1.11	0.73	0.16	0.44	1.06	0.263
**C18:1n9 cis/trans Oleic acid [mg/ml]**	1.53	0.54	0.58	3.14	1.87	0.76	0.95	4.44	0.010*
**C18:1 Trans vaccinic acid [mg/ml]**	0.14	0.04	0.06	0.29	0.17	0.06	0.09	0.33	0.009*
**C18:2n6c Linolenic acid [mg/ml]**	1.25	0.41	0.63	2.46	1.32	0.44	0.61	2.51	0.421
**C18:3n6 Gamma linolenic acid [mg/ml]**	0.01	0.01	0.00	0.04	0.01	0.01	0.00	0.04	0.213
**C18:3n3 Linolenic acid [mg/ml]**	0.05	0.03	0.01	0.16	0.06	0.03	0.02	0.17	0.010*
**C20:0 Arachidic acid [mg/ml]**	0.01	0.01	0.00	0.06	0.01	0.00	0.00	0.01	0.417
**C22:1 cis11- Eicosenic acid [mg/ml]**	0.01	0.00	0.01	0.03	0.02	0.01	0.01	0.03	0.026*
**C20:2 cis-11-Eicosadienoic acid [mg/ml]**	0.01	0.00	0.01	0.03	0.01	0.01	0.01	0.03	0.249
**C20:3n6 Eicosatrienoic acid [mg/ml]**	0.09	0.03	0.02	0.17	0.10	0.04	0.03	0.18	0.087
**C20:4n6 Arachidonic acid [mg/ml]**	0.44	0.14	0.21	0.85	0.47	0.15	0.20	0.73	0.322
**C20:5n3 EPA [mg/ml]**	0.06	0.05	0.01	0.30	0.07	0.04	0.02	0.22	0.723
**C22:0 Behenic acid [mg/ml]**	0.01	0.00	0.00	0.02	0.01	0.00	0.00	0.01	0.445
**C22:5w3 (docosapentaenate) [mg/ml]**	0.04	0.01	0.02	0.08	0.05	0.02	0.02	0.11	0.116
**C22:6n3 DHA [mg/ml]**	0.16	0.06	0.06	0.38	0.17	0.06	0.07	0.43	0.213
**LTX A4 5S, 6R, 15R [µg/ml]**	1.15	1.11	0.07	5.53	1.72	2.53	0.13	12.26	0.456
**LTX A4 5S, 6R [µg/ml]**	1.32	1.32	0.07	7.08	1.78	2.45	0.05	12.45	0.597

(BPH, benign prostatic hyperplasia; MetS, metabolic syndrome; n, number; X, arithmetic mean; SD, standard deviation; Min, mnimum; Max, maximum; p, statistical significance; WC, waist circumference; BMI, Body Mass Index; TG, triglyceride; TCh, total cholesterol; LDL, low-density lipoprotein; HDL, high-density lipoprotein; FPG, fasting plasma glucose; HOMA, insulin resistance; TT, total testosterone; TF, free testosterone; SHBG, sex hormone binding globulin; E2, estradiol; DHEAs, dehydroepiandrosterone sulfate; LH, luteinizing hormone; IGI-1, insulin like grow factor-1; I, insulin; *, statistical significant parameter)

The study also involved statistical analysis of the correlations between anthropometric, metabolic (Table 2), and hormonal parameters (Table 3) in BPH patients both with and without MetS. In the patients with MetS, serum TG levels correlated positively with the levels of the following acids: C12:0, C14:0, C15:0, C16:0, C16:1, C17:0, C18:0, C18:1n9, C18:1, C18:2n6, C18:3n6, C18:3n3, C22:1, C20:2, C20:3n6, C20:4n6, C22:5w3, C22:6n3. Similar correlations were observed for the patients without MetS, except for C17:0, which correlated negatively, and C15:0, C18:3n3, C20:4n6, C22:6n3, which did not show any correlation. HDL levels in the MetS patients correlated positively only with C10:0 and negatively with C18:1n9. Some of the acids correlated also with LDL levels, which were lower in the patients with MetS, but the difference was not statistically significant.

**Table 2:** Correlations between anthropometric and metabolic parameters and the levels of polyunsaturated fatty acids in BPH patients with and without MetS.

**Variables**		**Patients with BPH and METS**								**Patients with BPH and without METS**							
		**Age**	**Body weight**	**WC**	**TG**	**TCh**	**HDL**	**LDL**	**FPG**	**Age**	**Body weight**	**WC**	**TG**	**TCh**	**HDL**	**LDL**	**FPG**
**C10:0 Capric acid**	P	-0.317*	0.312*	0.257	-0.026	0.046	0.288*	-0.008	-0.071	-0.195	0.086	-0.131	0.110	0.001	-0.114	0.027	-0.131
	p	**0.022**	**0.024**	0.065	0.852	0.748	0.039	0.958	0.618	0.058	0.410	0.205	0.291	0.996	0.273	0.796	0.205
**C12:0 Lauric acid**	P	0.027	-0.027	-0.024	0.467*	0.384*	-0.128	0.241	0.152	-0.225*	0.081	-0.064	0.310*	0.049	-0.225*	0.070	0.022
	p	0.849	0.851	0.863	**<0.001**	**0.005**	0.366	0.088	0.282	**0.029**	0.436	0.541	**0.002**	0.638	**0.029**	0.506	0.833
**C14:0 Myristic acid**	P	-0.041	0.078	0.115	0.697*	0.548*	-0.118	0.304*	0.314*	-0.378*	0.150	-0.062	0.523*	0.136	-0.189	0.097	0.055
	p	0.775	0.581	0.419	**<0.001**	**<0.001**	0.406	**0.030**	**0.024**	**<0.001**	0.148	0.551	**<0.001**	0.188	0.067	0.351	0.595
**C14:1 Myristolenic acid**	P	0.260	-0.028	-0.007	0.327*	0.136	-0.046	-0.041	0.260	-0.150	0.010	-0.107	0.210*	-0.072	-0.190	-0.045	-0.109
	p	0.066	0.847	0.959	**0.019**	0.340	0.750	0.776	0.065	0.146	0.925	0.302	0.041	0.489	0.065	0.670	0.291
**C15:0 Pentadecanoid acid**	P	-0.096	0.016	-0.038	0.426*	0.439*	-0.033	0.291*	0.127	-0.124	0.038	-0.181	0.099	0.103	-0.061	0.101	0.065
	p	0.498	0.912	0.788	**0.002**	**0.001**	0.817	**0.038**	0.368	0.230	0.715	0.080	0.340	0.321	0.557	0.332	0.529
**C15:1 cis-10-pentadecanoid acid**	P	0.287*	0.010	0.041	0.086	-0.030	-0.111	-0.053	0.006	-0.090	-0.087	-0.105	-0.006	0.042	-0.050	0.069	0.002
	p	**0.039**	0.944	0.774	0.545	0.834	0.432	0.714	0.964	0.392	0.409	0.319	0.956	0.689	0.638	0.516	0.988
**C16:0 Palmitic acid**	P	-0.151	0.198	0.206	0.736*	0.600*	-0.210	0.383*	0.418*	-0.323*	0.098	-0.118	0.477*	0.229*	-0.148	0.192	-0.040
	p	0.286	0.158	0.142	**<0.001**	**<0.001**	0.136	**0.006**	**0.002**	**0.001**	0.347	0.256	**<0.001**	**0.025**	0.151	0.063	0.697
**C16:1 Palmitoleic acid**	P	-0.157	0.232	0.282*	0.773*	0.539*	-0.227	0.271	0.523*	-0.306*	0.143	-0.039	0.577*	0.139	-0.230*	0.111	0.015
	p	0.266	0.098	0.043	**<0.001**	**<0.001**	0.106	0.054	**<0.001**	**0.003**	0.166	0.710	**<0.001**	0.179	**0.025**	0.285	0.884
**C17:0 Heptadecanoid acid**	P	-0.195	0.079	<0.001	0.477*	0.414*	0.006	0.239	0.199	-0.133	0.041	-0.230*	0.146	0.070	-0.083	0.064	0.056
	p	0.166	0.575	0.998	**<0.001**	**0.002**	0.964	0.091	0.157	0.200	0.690	**0.025**	0.159	0.502	0.427	0.542	0.591
**C18:0 Stearic acid**	P	-0.169	0.160	0.136	0.456*	0.521*	-0.128	0.419*	0.279*	-0.328*	0.055	-0.184	0.269*	0.198	-0.005	0.145	-0.084
	p	0.230	0.256	0.336	**0.001**	**<0.001**	0.364	**0.002**	**0.046**	**0.001**	0.597	0.074	**0.008**	0.055	0.962	0.163	0.418
**C18:1n9 cis /trans Oleic acid**	P	-0.179	0.223	0.258	0.786*	0.514*	-0.274*	0.256	0.471*	-0.283*	0.112	-0.051	0.606*	0.111	-0.270*	0.085	-0.074
	p	0.203	0.113	0.065	**<0.001**	**<0.001**	**0.050**	0.069	**<0.001**	**0.005**	0.278	0.625	**<0.001**	0.284	**0.008**	0.414	0.479
**C18:1 trans vaccinic acid**	P	-0.257	0.200	0.216	0.684*	0.528*	-0.201	0.310*	0.456*	-0.189	0.042	-0.158	0.495*	0.155	-0.202*	0.132	-0.077
	p	0.066	0.154	0.124	**<0.001**	**<0.001**	0.152	0.027	**0.001**	0.066	0.688	0.126	**<0.001**	0.134	**0.050**	0.204	0.459
**C18:2n6c Linolenic acid**	P	-0.184	0.070	0.073	0.418*	0.569*	-0.176	0.511*	0.121	-0.237*	0.019	-0.191	0.302*	0.373*	-0.035	0.337*	-0.030
	p	0.191	0.622	0.605	**0.002**	**<0.001**	0.211	**<0.001**	0.391	**0.021**	0.853	0.064	**0.003**	**<0.001**	0.740	**0.001**	0.773
**C18:3n6 gamma linolenic acid**	P	-0.129	0.281*	0.206	0.516*	0.261	-0.160	0.101	0.287*	-0.362*	0.213*	-0.061	0.279*	0.216*	0.012	0.157	0.001
	p	0.361	**0.044**	0.143	**<0.001**	0.062	0.258	0.480	**0.039**	**<0.001**	**0.039**	0.560	**0.006**	**0.036**	0.906	0.130	0.989
**C18:3n3 linolenic acid**	P	-0.136	0.048	0.067	0.587*	0.524*	-0.207	0.357*	0.243	-0.123	-0.103	-0.191	0.463*	0.144	-0.149	0.106	0.064
	p	0.338	0.737	0.639	**<0.001**	**<0.001**	0.142	**0.010**	0.082	0.235	0.322	0.063	<0.001	0.164	0.148	0.308	0.536
**C20:0 Arachidic acid**	P	-0.024	0.161	0.198	0.327*	0.235	-0.104	0.124	0.241	-0.177	-0.190	-0.259*	-0.031	-0.257*	-0.027	-0.260*	0.107
	p	0.871	0.281	0.183	**0.025**	0.111	0.487	0.411	0.103	0.139	0.113	0.029	0.800	**0.030**	0.826	**0.030**	0.374
**C22:1 cis11- eicosenic acid**	P	-0.215	0.161	0.141	0.586*	0.535*	-0.057	0.322*	0.411*	-0.160	-0.054	-0.268*	0.419*	-0.009	-0.134	-0.049	-0.002
	p	0.125	0.255	0.320	**<0.001**	**<0.001**	0.688	**0.021**	**0.003**	0.122	0.605	**0.009**	**<0.001**	0.933	0.195	0.637	0.988
**C20:2 cis-11-eicosadienoic acid**	P	-0.048	0.043	0.123	0.474*	0.349*	-0.166	0.211	0.299*	-0.218*	0.053	-0.184	0.281*	0.296*	-0.076	0.277*	-0.012
	p	0.737	0.767	0.390	**<0.001**	**0.012**	0.245	0.142	**0.033**	**0.037**	0.616	0.079	**0.007**	**0.004**	0.474	**0.008**	0.912
**C20:3n6 eicosatrienoic acid**	P	-0.311*	0.342*	0.246	0.299*	0.344*	-0.152	0.331*	0.188	-0.282*	0.252*	-0.060	0.241*	0.263*	-0.174	0.287*	-0.063
	p	**0.025**	**0.013**	0.079	**0.031**	**0.013**	0.281	**0.018**	0.181	**0.006**	**0.014**	0.564	**0.018**	**0.010**	0.092	**0.005**	0.542
**C20:4n6 Arachidonic acid**	P	-0.309*	0.244	0.077	0.283*	0.416*	0.171	0.304*	0.200	-0.167	0.144	-0.120	0.073	0.222*	0.099	0.173	-0.119
	p	**0.026**	0.081	0.588	**0.042**	**0.002**	0.226	**0.030**	0.154	0.105	0.163	0.248	0.485	**0.031**	0.339	0.096	0.252
**C20:5n3 EPA**	P	-0.064	0.039	-0.090	0.181	0.323*	0.047	0.281*	0.070	0.005	-0.041	-0.079	-0.101	-0.064	0.226*	-0.139	0.187
	p	0.653	0.784	0.526	0.199	**0.020**	0.742	**0.046**	0.622	0.963	0.695	0.445	0.328	0.537	**0.028**	0.180	0.070
**C22:5w3 (docosapentaenate)**	P	-0.148	0.065	-0.034	0.453*	0.503*	-0.052	0.380*	0.264	-0.265*	-0.010	-0.268*	0.299*	0.262*	-0.055	0.230*	0.044
	p	0.294	0.647	0.810	**0.001**	**<0.001**	0.715	**0.006**	0.059	**0.009**	0.921	**0.009**	**0.003**	**0.010**	0.596	**0.025**	0.674
**C22:6n3 DHA**	P	-0.119	0.061	0.002	0.303*	0.391*	-0.150	0.346*	0.176	-0.038	0.021	-0.118	0.023	0.078	0.006	0.070	0.101
	p	0.400	0.666	0.990	**0.029**	**0.004**	0.287	**0.013**	0.212	0.717	0.840	0.255	0.826	0.450	0.951	0.500	0.330
**LTX A4 5S, 6R, 15R**	P	-0.267	0.220	0.182	0.102	-0.040	-0.056	-0.076	-0.066	-0.295*	0.297*	0.242*	0.007	0.067	-0.125	0.111	-0.015
	p	0.055	0.117	0.196	0.473	0.780	0.694	0.598	0.644	**0.004**	**0.004**	**0.019**	0.947	0.520	0.228	0.289	0.883
**LTX A4 5S, 6R**	P	-0.306*	0.218	0.185	0.037	-0.034	-0.032	-0.029	-0.198	-0.239*	0.261*	0.241*	-0.005	0.048	-0.111	0.088	-0.016
	p	**0.028**	0.121	0.190	0.797	0.812	0.822	0.837	0.159	**0.020**	**0.011**	**0.019**	0.961	0.648	0.287	0.402	0.876

(BPH, benign prostatic hyperplasia; METS, metabolic syndrome; p, statistical significance; P, correlation coefficient; WC, waist circumference; BMI, Body Mass Index; TG, triglyceride; TCh, total cholesterol; LDL, low-density lipoprotein; HDL, high-density lipoprotein; FPG, fasting plasma glucose; *, statistical significant parameter)

**Table 3:** Correlations between hormonal parameters and the levels of polyunsaturated fatty acids in BPH patients with and without MetS.

**Variables**		**Patients with BPH and METS**								**Patients with BPH and without METS**							
		**DHEA**	**E2**	**SHBG**	**LH**	**TT**	**TF**	**IGF1**	**I**	**DHEA**	**E2**	**SHBG**	**LH**	**TT**	**TF**	**IGF1**	**I**
**C10:0 Capric acid**	P	0.055	0.132	-0.273	-0.014	-0.151	0.367*	-0.263	0.036	-0.038	0.039	-0.125	-0.212*	0.010	-0.004	-0.024	-0.128
	p	0.696	0.350	0.050	0.920	0.285	**0.007**	0.059	0.825	0.714	0.706	0.228	**0.039**	0.925	0.969	0.821	0.256
**C12:0 Lauric acid**	P	0.178	-0.095	-0.230	0.030	-0.243	-0.153	-0.194	0.140	0.042	0.111	-0.221*	-0.119	-0.095	-0.031	-0.030	0.111
	p	0.207	0.501	0.101	0.833	0.083	0.279	0.168	0.383	0.689	0.288	**0.032**	0.255	0.360	0.767	0.775	0.329
**C14:0 Myristic acid**	P	0.068	-0.046	-0.378*	-0.003	-0.267	0.082	-0.213	0.062	0.125	0.147	-0.272*	-0.096	0.004	0.088	0.063	0.191
	p	0.630	0.746	**0.006**	0.986	0.056	0.562	0.129	0.701	0.229	0.155	**0.008**	0.356	0.973	0.396	0.541	0.090
**C16:0 Palmitic acid**	P	0.183	-0.032	-0.329*	-0.025	-0.308*	0.160	-0.137	0.049	0.166	0.058	-0.141	-0.091	0.125	0.150	0.153	0.060
	p	0.193	0.820	**0.017**	0.859	**0.026**	0.256	0.332	0.761	0.108	0.579	0.174	0.380	0.227	0.147	0.138	0.598
**C18:0 Stearic acid**	P	0.304*	-0.062	-0.283*	-0.123	-0.375*	0.004	-0.158	-0.023	0.086	-0.024	-0.100	-0.070	0.077	0.089	0.220*	0.024
	p	**0.028**	0.660	**0.042**	0.385	**0.006**	0.975	0.263	0.885	0.409	0.819	0.337	0.500	0.457	0.389	**0.032**	0.832
**C18:1n9 cis/trans Oleic acid**	P	0.196	0.015	-0.305*	0.048	-0.273	0.231	-0.149	0.059	0.170	0.096	-0.132	-0.136	0.076	0.115	0.180	0.045
	p	0.165	0.915	**0.028**	0.733	0.050	0.100	0.293	0.714	0.099	0.355	0.202	0.189	0.464	0.267	0.082	0.694
**C18:1 trans vaccinic acid**	P	0.382*	-0.025	-0.276*	-0.006	-0.256	0.161	-0.145	0.053	0.210*	0.084	-0.037	-0.068	0.098	0.164	0.266*	0.028
	p	**0.005**	0.861	**0.048**	0.968	0.067	0.254	0.304	0.744	**0.042**	0.416	0.725	0.515	0.347	0.113	**0.009**	0.807
**C18:2n6c Linolenic acid**	P	0.128	-0.015	-0.283*	-0.150	-0.284*	0.080	-0.129	-0.051	0.072	-0.009	-0.080	-0.090	0.059	0.062	0.310*	-0.022
	p	0.365	0.915	**0.042**	0.290	**0.041**	0.573	0.363	0.751	0.487	0.931	0.443	0.385	0.573	0.548	**0.002**	0.848
**C18:3n6 gamma linolenic acid**	P	0.138	-0.029	-0.283*	0.056	-0.225	0.117	-0.262	-0.020	-0.005	0.127	-0.213*	0.003	0.037	0.073	-0.004	0.053
	p	0.328	0.839	**0.042**	0.693	0.109	0.411	0.060	0.900	0.965	0.220	**0.038**	0.978	0.720	0.484	0.967	0.643
**C18:3n3 linolenic acid**	P	0.020	-0.002	-0.314*	-0.131	-0.289*	0.117	-0.122	0.041	0.002	0.009	-0.130	-0.111	-0.176	-0.040	0.213*	0.009
	p	0.890	0.987	**0.024**	0.356	**0.038**	0.409	0.390	0.798	0.982	0.929	0.208	0.283	0.088	0.700	**0.038**	0.933
**C20:0 Arachidic acid**	P	-0.109	0.183	-0.244	-0.013	-0.322*	<0.001	-0.155	0.173	-0.069	-0.044	-0.122	0.025	-0.139	-0.098	-0.077	-0.131
	p	0.467	0.218	0.099	0.932	**0.027**	0.998	0.298	0.306	0.569	0.717	0.310	0.834	0.249	0.418	0.524	0.311
**C22:1 cis11- eicosenic acid**	P	0.316*	0.082	-0.243	-0.082	-0.285*	0.193	-0.189	0.058	0.210*	-0.024	-0.129	0.016	-0.026	0.121	0.247*	-0.042
	p	**0.023**	0.564	0.083	0.564	**0.040**	0.171	0.181	0.719	**0.041**	0.816	0.213	0.881	0.805	0.242	**0.016**	0.710
**C20:2 cis-11-eicosadienoic acid**	P	0.317*	-0.138	-0.234	-0.049	-0.256	-0.012	-0.188	0.032	0.192	-0.032	-0.062	-0.084	0.082	0.162	0.217*	0.113
	p	**0.023**	0.335	0.099	0.734	0.070	0.931	0.188	0.842	0.067	0.765	0.557	0.428	0.437	0.123	**0.038**	0.326
**C20:3n6 eicosatrienoic acid**	P	0.294*	0.048	-0.307*	-0.080	-0.366*	0.021	-0.155	0.044	0.117	-0.029	-0.126	-0.106	0.057	0.145	0.166	0.107
	p	0.035	0.734	**0.027**	0.572	**0.008**	0.885	0.274	0.782	0.258	0.783	0.225	0.309	0.583	0.162	0.109	0.344
**C20:4n6 Arachidonic acid**	P	0.305*	0.056	-0.284*	-0.181	-0.307*	0.132	-0.132	0.018	0.034	0.036	-0.089	-0.027	0.081	0.005	0.205*	-0.049
	p	**0.028**	0.693	**0.042**	0.200	**0.027**	0.351	0.352	0.912	0.744	0.731	0.390	0.793	0.438	0.958	**0.047**	0.663
**C20:5n3 EPA**	P	-0.038	0.143	-0.089	-0.349*	-0.172	0.061	-0.103	0.068	-0.025	0.008	-0.198	0.291*	-0.113	-0.046	0.014	0.057
	p	0.789	0.311	0.531	**0.011**	0.224	0.668	0.468	0.671	0.808	0.941	0.054	**0.004**	0.276	0.655	0.896	0.618
**C22:5w3 (docosapentaenate)**	P	0.241	0.044	-0.082	-0.172	-0.169	0.179	-0.207	0.024	0.131	<0.001	-0.092	-0.052	0.078	0.120	0.139	-0.036
	p	0.086	0.755	0.562	0.221	0.230	0.205	0.140	0.880	0.206	0.999	0.376	0.615	0.451	0.248	0.180	0.753
**C22:6n3 DHA**	P	0.236	0.016	-0.081	-0.275*	-0.152	0.096	-0.013	-0.061	-0.086	-0.028	-0.192	0.174	-0.150	-0.095	0.208*	0.076
	p	0.092	0.910	0.567	**0.048**	0.282	0.498	0.926	0.705	0.407	0.786	0.062	0.091	0.146	0.359	**0.043**	0.500
**LTX A4 5S, 6R, 15R**	P	-0.048	0.115	-0.221	0.035	-0.148	-0.041	-0.097	0.422*	-0.034	-0.077	-0.351*	-0.136	-0.202	-0.062	-0.046	0.009
	p	0.738	0.417	0.115	0.807	0.294	0.775	0.495	**0.006**	0.745	0.462	**0.001**	0.190	0.051	0.551	0.660	0.935
**LTX A4 5S, 6R**	P	-0.083	0.055	-0.259	0.065	-0.153	-0.083	-0.092	0.239	-0.007	-0.025	-0.348*	-0.130	-0.184	-0.031	-0.048	0.045
	p	0.559	0.701	0.064	0.647	0.279	0.560	0.515	0.132	0.945	0.808	**0.001**	0.213	0.076	0.767	0.648	0.691

(BPH, benign prostatic hyperplasia; METS, metabolic syndrome; p, statistical significance; P, correlation coefficient; TT, total testosterone; TF, free testosterone; SHBG, sex hormone binding globulin; E2, estradiol; DHEAs, dehydroepiandrosterone sulfate; LH, luteinizing hormone; IGI-1, insulin like grow factor-1; I, insulin; *, statistical significant parameter)

We also analyzed the relationship between the levels of fatty acids and diagnostic parameters for MetS (Table 4). Particular abnormalities were associated with single changes in the levels of fatty acids. In the diabetic patients, changes in the levels of pentadecanoic acid, heptadecanoic acid, trans-vaccenic acid, and cis-11-eicosenoic acid were demonstrated. Changes in the levels of arachidic acid―5(S), 6(R), 15(R)-LXA4 and 5(S), 6(R)- LXA4―were noted in the patients with central obesity.

**Table 4:** Relationships between the levels of fatty acids and diagnostic MetS parameters in patients with BPH.

**Variables**	**WC** **< 94 cm** **n=53**		**WC** **≥94 cm** **n=101**		**p**	**No-statin drugs** **n=106**		**Statin drugs n=48**		**p**	**No- diabetes n=116**		**Diabetes** **n=38**		**p**	**No-hypertension n=97**		**Hypertension n=57**		**p**
	**X**	**SD**	**X**	**SD**		**X**	**SD**	**X**	**SD**		**X**	**SD**	**X**	**SD**		**X**	**SD**	**X**	**SD**	
**C15:0** **Pentadecanoid acid** [mg/ml]	0.016	0.005	0.016	0.006	0.627	0.015	0.006	0.017	0.005	0.097	0.015	0.005	0.018	0.007	0.032*	0.016	0.006	0.015	0.006	0.287
**C17:0** **Heptadecanoid acid** [mg/ml]	0.019	0.005	0.019	0.007	0.488	0.019	0.006	0.020	0.006	0.092	0.018	0.005	0.022	0.008	0.014*	0.019	0.006	0.019	0.006	0.342
**C18:1** **Trans vaccinic acid** [mg/ml]	0.148	0.049	0.150	0.051	0.885	0.147	0.052	0.155	0.046	0.238	0.143	0.045	0.170	0.061	0.033*	0.156	0.053	0.139	0.045	0.081
**C20:0** **Arachidic acid** [mg/ml]	0.009	0.008	0.007	0.002	0.044*	0.008	0.006	0.007	0.002	0.414	0.008	0.006	0.007	0.002	0.325	0.007	0.002	0.009	0.008	0.135
**C22:1** **cis11- eicosenic acid** [mg/ml]	0.016	0.005	0.015	0.005	0.588	0.015	0.005	0.016	0.005	0.105	0.015	0.005	0.017	0.006	0.014*	0.016	0.005	0.014	0.005	0.081
**LTX A4 5S, 6R, 15R** [µg/ml]	0.951	0.988	1.562	2.019	0.031*	1.177	1.407	1.762	2.344	0.202	1.353	1.777	1.372	1.741	0.745	1.476	2.023	1.143	1.139	0.693
**LTX A4 5S, 6R** [µg/ml]	1.048	1.097	1.704	2.050	0.026*	1.355	1.603	1.772	2.198	0.332	1.456	1.636	1.572	2.303	0.887	1.548	1.996	1.368	1.423	0.953

(X, arithmetic mean; SD, standard deviation; p, statistical significance; n, number; WC, waist circumference; *, statistical significant parameter)

## DISCUSSION

Analysis of fatty acids can provide valuable information on MetS in BPH patients. To date, pieces of research in this field have assessed the serum levels of fatty acids in patients with MetS with regard to various diseases, such as chronic renal disease [19], systemic lupus [20], arterial stiffness [21], and non-alcoholic fatty liver disease (NAFLD) [22]. Studies of prostatic diseases have involved comparative analysis of biochemical and metabolic parameters and the levels of fatty acids in BPH and prostate cancer [23–26]. However, the relationship between the levels of fatty acids and MetS in BPH have not so far been described.

Our investigation of metabolic and hormonal parameters in BPH patients with regard to concomitant MetS revealed statistically significant differences in terms of SHBG and MetS parameters. Produced mainly in the liver, SHBG is a glycoprotein that binds and transports sex hormones [27]. This protein has the strongest affinity for dihydrotestosterone (DHT), and influences its bioavailability. After 40 years of age, the level of SHBG gradually raises by approximately 1% per year, thus leading to a decline in testosterone activity. It can increase hypogonadism, which shows the relationship between SHBG and BPH. Another parameter whose levels statistically significantly differed between the groups was IGF-1. This hormone, produced in the liver, is similar to insulin in structure. It is believed to be involved in initiating signaling pathways associated with the growth and proliferation of the prostate cells [28,29]. Rył et al. [29] reported that the levels of IGF-1 in BPH patients without MetS correlated with the parameters of their lipid profiles. Moreover, an excess of insulin in the blood boosts the activity of the sympathetic nervous system, thus increasing the prostate smooth muscle tone [30,31]. Disturbances of insulin secretion have an impact on the development of BPH, however the nature of this relationship has not been fully elucidated [32,33]. Our study, on the other hand, confirmed that in the group without MetS, the level of IGF-1 correlated positively with the levels of fatty acids, namely stearic acid (C18:0), trans vaccenic acid (C18:1), linoleic acid (C18:2n6c), linolenic acid (C18:3n3), eicosanoic acid (C22:1cis11), eicosadienoic acid (C22:2cis11), arachidonic acid (C20:4n6), and docosahexaenoic acid (DHA) (C22:6n3). We also noted substantially higher serum levels of selected saturated fatty acids (SFA): C14:0, C15:0, C16:0, C17:0; monounsaturated fatty acids: C14:1, C16:1, C18:1n9, C18:1; and polyunsaturated fatty acids: C18:3n6 in the patients with MetS compared with those without this syndrome. This observation suggests that elevated levels of fatty acids may contribute to metabolic disorders in BPH patients. Furthermore, we demonstrated correlations between biochemical and hormonal parameters and fatty acids. Fatty acids in serum have a profound effect on metabolism. Plasma TG levels can rise depending on the levels of saturated fatty acids, and drop contingent on the levels of polyunsaturated fatty acids. The population study conducted by Lopez-Alvarenga et al. [34] revealed a strong association between serum fatty acid and TG levels. Polyunsaturated fatty acids (20:3ω6, 20:4ω6, 20:5 ω3, 22:5ω3) correlated negatively with serum TG levels, while palmitic acid (C16:0) and linolenic acid (C18:3ω3) correlated positively. This study also confirmed that higher serum levels of saturated fatty acids contribute to the development of MetS, which is supported by Warensjö et al. [35], who carried out the population-based cohort study of aging men and found that serum fatty acid levels were related to MetS.

In our study, the majority of fatty acids correlated positively with their serum lipid fractions in MetS patients. A strong relationship between fatty acid and serum TG levels in patients with BPH indicates a potential role of these acids in the development of MetS. Similarly to us, Lopez-Alvarenga et al. [34] did not notice any connection between fatty acids, the HOMA-IR index, and insulin levels in the patients with and without MetS. Kotronen et al. [36] analyzed the levels of fatty acids in serum lipid fractions. These researchers provided evidence for the correlations between the selected fatty acids and the HOMA-IR index. They also indicated that the levels of specific fatty acids (mainly C16:0 and C18:1) are precise diagnostic markers of insulin resistance. In our study, the MetS patients had higher insulin levels and the HOMA-IR index, but these results were not statistically significant. We did not note any link between fatty acid levels and the HOMA-IR index in the patients with or without MetS. Insulin levels positively correlated only with the isomers of lipoxin A4 (LXA4)―5(S), 6(R), 15(R)-LXA4―in the patients with MetS. Novgorodtseva et al. [37] noticed the increased levels of polyunsaturated fatty acids, and the decreased levels of saturated fatty acids and proinflammatory eicosanoids in MetS patients with disturbed glucose-insulin homeostasis and those with insulin resistance. Based on experimental animal models, the authors demonstrated that LXA4 may be a factor that alleviates inflammatory response associated with the accumulated adipose tissue, and thus can prevent insulin resistance and MetS [38,39]. The study of the Chinese middle-age population [40], on the other hand, revealed that people with lower serum LXA4 levels were more likely to go down with MetS than those with higher lipoxin levels. Our observation was different, which may arise from the differing methods employed to determine serum LXA4 levels. Pickens et al. [41] reported elevated LXA4 levels in obese people, which corresponds with our findings. We also found that in MetS patients, LXA4 levels correlated not only with the HOMA-IR index and insulin levels, but also with anthropometric parameters. In the group of patients without MetS, we observed statistically significantly lower values of body mass and waist circumference, which correlated with the values obtained for 5(S), 6(R), 15(R)-LXA4. Obesity and aging are known to be factors in chronic mild inflammation, which additionally contributes to type 2 diabetes and hypertension. Adipose tissue, regarded as an additional organ showing immune activity, can entail chronic mild inflammation, thus promoting the development of insulin resistance, type 2 diabetes, and MetS [42]. Moreover, it is difficult to unambiguously determine secondary effects of changes in fatty acids and their connection with BPH. The available literature shows that both BPH and metabolic disorders may be related to the activity of the inflammatory process. In our further research, we plan to analyze the activity of the inflammatory process in the prostate cells of men without BPH [25,43].

The contribution of the remaining hormones to BPH has not been clearly defined. Many authors have examined the effects of testosterone (T), DHT, and estrogen (E) upon BPH and LUTS. Their results, however, are not consistent [44–46].

The studies conducted so far have not demonstrated an increase in the risk of BPH or LUTS as a result of elevated serum testosterone levels. Although replacement testosterone therapy may cause exacerbation of these conditions [47,48], higher serum testosterone levels cannot be regarded as the cause of BPH. The response of the prostate to estrogen depends on many variables, such as a dose, the time of exposure, and the presence of androgens [49]. Higher doses of estrogen can even lead to prostate cancer [50]. The relationship between the levels of dehydroepiandrosterone (DHEA) and dehydroepiandrosterone sulphate (DHEAS) and the levels of testosterone and estradiol explains their contribution to the development of LUTS and BPH [51].

A conclusion that can be drawn from the study is that changes in the levels of fatty acids influence biochemical and hormonal parameters in BPH patients. Analysis of patients with BPH showed that MetS contributed to changes in their levels of polysaturated fatty acids (particular abnormalities were associated with single changes). The levels of pentadecanoic, heptadecanoic, and cis-11-eicosenoic acids were higher in diabetic patients, however even within this group differences were observed. It is also worth emphasizing that changes in the levels of polysaturated fatty acids, suggesting atherosclerotic changes, were demonstrated in all patients involved in the study―both with and without MetS. Our findings indicate the necessity for further investigation concerning the levels of fatty acids and their impact on the development of MetS, as well as the course and clinical picture of BPH. It would be reasonable to detect early symptoms of BPH, especially in patients with diagnosed MetS, as the prevention of urological surgery.

## MATERIALS AND METHODS

The study involved 154 men with BPH, aged between 46 and 80 years (mean ± SD: 67.15 ± 7.10), qualified for transurethral resection of the prostate (TURP) in the Clinic of Urology and Urologic Oncology, Pomeranian Medical University in Szczecin, due to symptomatic BPH. Diagnosis was based on a high International Prostate Symptom Score (IPSS), a decreased maximum flow rate (Qmax), long lasting symptoms BPH or urinary retention. Patients with cancer diseases, active alcohol disease, and thyroid disease were excluded from the study. Neither prostate volume nor PSA level were routinely measured in patients before admitting them for surgery. Therefore, we decided not to compare these parameters between the study group and the control group.

The study was approved by the Bioethical Commission of the Pomeranian Medical University in Szczecin (approval number KB-0012/123/14). The participants gave informed written consent to take part in it. Anthropometric parameters, including weight, height, age, and waist circumference, were measured for all patients. Additionally, the participants completed a questionnaire concerning demographic data and chronic diseases. The men were divided into two groups: those without (n = 101) and those with MetS (n = 53). MetS was diagnosed on the basis of the International Diabetes Federation (IDF) 2005 criteria [52]. The patients included in the MetS group had central obesity ≥ 94cm, and at least two of the following abnormalities: triglycerides ≥ 150mg/dl or treatment for dyslipidemia; HDL cholesterol < 40mg/dl or treatment for dyslipidemia; blood pressure ≥ 130/85mmHg or treatment for hypertension; fasting glycemia ≥ 100mg/dl or treatment for type 2 diabetes. All components of MetS were considered individually.

The body mass index (BMI) was determined: overweight was diagnosed for BMI between 25 and 29.99 kg/m2, and obesity for BMI ≥ 30 kg/m2. Additionally, the insulin resistance (HOMA-IR) index was calculated for nondiabetic patients according to the formula: fasting glucose (mmol/l) × fasting insulin (μU/ml) / 22.5 [53].

### Blood serum analysis

9-ml blood samples were taken for laboratory analysis from a cubital vein on an empty stomach between 7.30 am and 9.00 am. The blood was collected using tubes with clot activator and gel separator, and then centrifuged. The serum levels of fasting plasma glucose (FPG) in nondiabetic men, total cholesterol (TCh), low density lipoprotein (LDL), highdensity lipoprotein (HDL) cholesterol, and triglycerides were determined using a spectrophotometric method with commercial reagent kits (Biolabo, Aqua-Med, Łódź, Poland).

The ELISA method with commercial reagent kits (DRG International, Germany) was employed to determine the serum levels of the selected hormones—total testosterone (TT), free testosterone (FT), insulin (I), DHEAS, estradiol (E2), luteinizing hormone (LH), insulin-like growth factor 1 (IGF-1) and sex hormone-binding globulin (SHBG).

### Isolation of fatty acids

Serum was obtained from blood clots centrifuged for 10 min at 1200 G. Fatty acids were extracted by the method of Folch [54]. A 0.5 mL serum sample was saponified with 1 mL of 2 mol/L KOH methanolic solution at 70 °C for 20 min, and then methylated with 2 mL of 14% boron trifluoride in methanol under the same conditions. Next, 2 mL of n-hexane and 10 mL of saturated NaCl solution were added. 1 mL n-hexane was taken for analysis.

### Analysis of fatty acid methyl esters

Gas chromatography was performed using the Agilent Technologies 7890A GC System (a SUPELCOWAX™ 10 Capillary GC Column (15 mm × 0.10 mm, 0.10 μm); Supelco, Bellefonte, PA, United States). The following chromatographic conditions were applied: the initial temperature was 60 °C for 0 min; it rose to 160 °C (0 min) at a rate of 40°C/min; next, it rose to 190 °C (0.5 min) at a rate of 30 °C/min, and next to 230 °C (2.6 min) at a rate of 30 °C/min. The whole analysis lasted approximately 8 min, and the gas flow rate was 0.8 mL/min with hydrogen used as the carrier gas. Fatty acids were identified by comparing their retention times with those of commercially available.

### Statistical analysis

Statistical analysis was performed using Statistica 12 software (StatSoft, Inc. Tulsa, OK, USA). The study sample was characterized by basic statistics (mean, standard deviation, minimum and maximum values). The normality of the distribution was assessed using the Shapiro-Wilk test. Student’s *t*-test and the Mann-Whitney *U* test were applied to determine differences between groups. Correlations between the quantitative variables were calculated using Pearson’s correlation coefficient. The level of significance was set at p ≤ 0.005.

### Limitations of the study

The limitation of the study is the fact that selected parameters were only assessed in BPH patients. This, however, resulted from the premises of our research―we had planned to analyze the levels of selected fatty acids exclusively in patients with BPH with regard to MetS, as BPH in men from the studied age bracket can be asymptomatic. It should be emphasized that all participants receiving treatment for BPH ― both the study group and the control group―were patients deviating from the healthy population. Another limitation is the lack of specific information regarding outcomes of TURP. This, however, was not the target of this study. Our findings motivate us to conduct further comparative analysis of the same parameters in patients without BPH.

## References

[1] Roehrborn C, McConnell J. (2002). Etiology, pathophysiology, epidemiology and natural history of benign prostatic hyperplasia. In: Walsh P, Retik A, Vaughan E, Wein A, editors. Campbell’s Urology. 8th ed. Philadelphia: Saunders, 1297–1336.

[2] Gacci M, Corona G, Vignozzi L, Salvi M, Serni S, De Nunzio C, Tubaro A, Oelke M, Carini M, Maggi M. Metabolic syndrome and benign prostatic enlargement: a systematic review and meta-analysis. BJU Int. 2015; 115:24–31. 10.1111/bju.12728 24602293

[3] Giles GG, Severi G, English DR, McCredie MR, MacInnis R, Boyle P, Hopper JL. Early growth, adult body size and prostate cancer risk. Int J Cancer. 2003; 103:241–45. 10.1002/ijc.10810 12455039

[4] Owens GK, Kumar MS, Wamhoff BR. Molecular regulation of vascular smooth muscle cell differentiation in development and disease. Physiol Rev. 2004; 84:767–801. 10.1152/physrev.00041.2003 15269336

[5] Rył A, Rotter I, Miazgowski T, Słojewski M, Dołęgowska B, Lubkowska A, Laszczyńska M. Metabolic syndrome and benign prostatic hyperplasia: association or coincidence? Diabetol Metab Syndr. 2015; 7:94. 10.1186/s13098-015-0089-1 26516352 PMC4625953

[6] De Nunzio C, Aronson W, Freedland SJ, Giovannucci E, Parsons JK. The correlation between metabolic syndrome and prostatic diseases. Eur Urol. 2012; 61:560–70. 10.1016/j.eururo.2011.11.013 22119157

[7] Russo GI, Cimino S, Fragalà E, Privitera S, La Vignera S, Condorelli R, Calogero AE, Chisari M, Castelli T, Favilla V, Morgia G. Relationship between non-alcoholic fatty liver disease and benign prostatic hyperplasia/lower urinary tract symptoms: new insights from an Italian cross-sectional study. World J Urol. 2015; 33:743–51. 10.1007/s00345-014-1392-4 25189458

[8] Gacci M, Vignozzi L, Sebastianelli A, Salvi M, Giannessi C, De Nunzio C, Tubaro A, Corona G, Rastrelli G, Santi R, Nesi G, Serni S, Carini M, Maggi M. Metabolic syndrome and lower urinary tract symptoms: the role of inflammation. Prostate Cancer Prostatic Dis. 2013; 16:101–06. 10.1038/pcan.2012.44 23165431

[9] Vignozzi L, Gacci M, Cellai I, Morelli A, Maneschi E, Comeglio P, Santi R, Filippi S, Sebastianelli A, Nesi G, Serni S, Carini M, Maggi M. PDE5 inhibitors blunt inflammation in human BPH: a potential mechanism of action for PDE5 inhibitors in LUTS. Prostate. 2013; 73:1391–402. 10.1002/pros.22686 23765639

[10] Vanella L, Russo GI, Cimino S, Fragalà E, Favilla V, Li Volti G, Barbagallo I, Sorrenti V, Morgia G. Correlation between lipid profile and heme oxygenase system in patients with benign prostatic hyperplasia. Urology. 2014; 83:1444.e7–13. 10.1016/j.urology.2014.03.007 24862399

[11] Ros E. Dietary cis-monounsaturated fatty acids and metabolic control in type 2 diabetes. Am J Clin Nutr. 2003; 78:617S–25S. 10.1093/ajcn/78.3.617S 12936956

[12] Marchioli R, Levantesi G, Macchia A, Maggioni AP, Marfisi RM, Silletta MG, Tavazzi L, Tognoni G, Valagussa F, and GISSI-Prevenzione Investigators. Antiarrhythmic mechanisms of n-3 PUFA and the results of the GISSI-Prevenzione trial. J Membr Biol. 2005; 206:117–28. 10.1007/s00232-005-0788-x 16456722

[13] Kobayashi N, Barnard RJ, Henning SM, Elashoff D, Reddy ST, Cohen P, Leung P, Hong-Gonzalez J, Freedland SJ, Said J, Gui D, Seeram NP, Popoviciu LM, et al. Effect of altering dietary omega-6/omega-3 fatty acid ratios on prostate cancer membrane composition, cyclooxygenase-2, and prostaglandin E2. Clin Cancer Res. 2006; 12:4662–70. 10.1158/1078-0432.CCR-06-0459 16899616 PMC3410648

[14] Nowak JZ. Anti-inflammatory pro-resolving derivatives of omega-3 and omega-6 polyunsaturated fatty acids. Postepy Hig Med Dosw. 2010; 64:115–32. 20354260

[15] Calder PC. Omega-3 fatty acids and inflammatory processes. Nutrients. 2010; 2:355–74. 10.3390/nu2030355 22254027 PMC3257651

[16] Ross DJ, Hough G, Hama S, Aboulhosn J, Belperio JA, Saggar R, Brian J. Van Lenten, Abbas A, Mansoureh E, Srinivasa R, Alan MF and Mohamad N. Proinflammatory highdensity lipoprotein results from oxidized lipid mediators in the pathogenesis of both idiopathic and associated types of pulmonary arterial hypertension. Pulm Circ. 2015; 5:640–48. 10.1086/683695 26697171 PMC4671738

[17] Bojić LA, McLaren DG, Harms AC, Hankemeier T, Dane A, Wang SP, Rosa R, Previs SF, Johns DG, Castro-Perez JM. Quantitative profiling of oxylipins in plasma and atherosclerotic plaques of hypercholesterolemic rabbits. Anal Bioanal Chem. 2016; 408:97–105. 10.1007/s00216-015-9105-4 26511226

[18] Bogatcheva NV, Sergeeva MG, Dudek SM, Verin AD. Arachidonic acid cascade in endothelial pathobiology. Microvasc Res. 2005; 69:107–27. 10.1016/j.mvr.2005.01.007 15896353

[19] Huang X, Sjögren P, Ärnlöv J, Cederholm T, Lind L, Stenvinkel P, Lindholm B, Risérus U, Carrero JJ. Serum fatty acid patterns, insulin sensitivity and the metabolic syndrome in individuals with chronic kidney disease. J Intern Med. 2014; 275:71–83. 10.1111/joim.12130 24011327

[20] Ormseth MJ, Swift LL, Fazio S, Linton MF, Raggi P, Solus JF, Oeser A, Bian A, Gebretsadik T, Shintani A, Stein CM. Free fatty acids are associated with metabolic syndrome and insulin resistance but not inflammation in systemic lupus erythematosus. Lupus. 2013; 22:26–33. 10.1177/0961203312462756 23060481 PMC3684362

[21] Kim OY, Lim HH, Lee MJ, Kim JY, Lee JH. Association of fatty acid composition in serum phospholipids with metabolic syndrome and arterial stiffness. Nutr Metab Cardiovasc Dis. 2013; 23:366–74. 10.1016/j.numecd.2011.06.006 21920716

[22] Maciejewska D, Drozd A, Ossowski P, Ryterska K, Jamioł-Milc D, Banaszczak M, Raszeja-Wyszomirska J, Kaczorowska M, Sabinicz A, Stachowska E. Fatty acid changes help to better understand regression of nonalcoholic fatty liver disease. World J Gastroenterol. 2015; 21:301–10. 10.3748/wjg.v21.i1.301 25574105 PMC4284349

[23] Mamalakis G, Kafatos A, Kalogeropoulos N, Andrikopoulos N, Daskalopulos G, Kranidis A. Prostate cancer vs hyperplasia: relationships with prostatic and adipose tissue fatty acid composition. Prostaglandins Leukot Essent Fatty Acids. 2002; 66:467–77. 10.1054/plef.2002.0384 12144866

[24] Christensen JH, Fabrin K, Borup K, Barber N, Poulsen J. Prostate tissue and leukocyte levels of n-3 polyunsaturated fatty acids in men with benign prostate hyperplasia or prostate cancer. BJU Int. 2006; 97:270–73. 10.1111/j.1464-410X.2006.05951.x 16430627

[25] Careaga VP, Sacca PA, Mazza ON, Scorticati C, Vitagliano G, Fletcher SJ, Maier MS, Calvo JC. Fatty acid composition of human periprostatic adipose tissue from argentine patients and its relationship to prostate cancer and benign prostatic hyperplasia. Research In Cancer and Tumor. 2015; 4:1–6. 10.5923/j.rct.20150401.01

[26] Giskeødegård GF, Hansen AF, Bertilsson H, Gonzalez SV, Kristiansen KA, Bruheim P, Mjøs SA, Angelsen A, Bathen TF, Tessem MB. Metabolic markers in blood can separate prostate cancer from benign prostatic hyperplasia. Br J Cancer. 2015; 113:1712–19. 10.1038/bjc.2015.411 26633561 PMC4702000

[27] Hammond GL. Diverse roles for sex hormone-binding globulin in reproduction. Biol Reprod. 2011; 85:431–41. 10.1095/biolreprod.111.092593 21613632 PMC4480437

[28] Rajaram S, Baylink DJ, Mohan S. Insulin-like growth factor-binding proteins in serum and other biological fluids: regulation and functions. Endocr Rev. 1997; 18:801–31. 9408744 10.1210/edrv.18.6.0321

[29] Rył A, Rotter I, Slojewski M, Dolegowska B, Grabowska M, Baranowska-Bosiacka I, Laszczynska M. Hormone concentration, metabolic disorders and immunoexpression of androgen and estrogen-alpha receptors in men with benign prostatic hyperplasia and testosterone deficiency syndrome. Folia Histochem Cytobiol. 2015; 53:227–35. 10.5603/fhc.a2015.0026 26400665

[30] Sarma AV, Parsons JK, McVary K, Wei JT. Diabetes and benign prostatic hyperplasia/lower urinary tract symptoms--what do we know? J Urol. 2009; 182:S32–37. 10.1016/j.juro.2009.07.088 19846144

[31] De Nunzio C, Aronson W, Freedland SJ, Giovannucci E, Parsons JK. The correlation between metabolic syndrome and prostatic diseases. Eur Urol. 2012; 61:560–70. 10.1016/j.eururo.2011.11.013 22119157

[32] Oelke M, Kirschner-Hermanns R, Thiruchelvam N, Heesakkers J. Can we identify men who will have complications from benign prostatic obstruction (BPO)? ICI-RS 2011. Neurourol Urodyn. 2012; 31:322–26. 10.1002/nau.22222 22415947

[33] Jangir RN, Jain GC. Diabetes mellitus induced impairment of male reproductive functions: a review. Curr Diabetes Rev. 2014; 10:147–57. 10.2174/1573399810666140606111745 24919656

[34] Lopez-Alvarenga JC, Ebbesson SO, Ebbesson LO, Tejero ME, Voruganti VS, Comuzzie AG. Polyunsaturated fatty acids effect on serum triglycerides concentration in the presence of metabolic syndrome components. The Alaska-Siberia Project. Metabolism. 2010; 59:86–92. 10.1016/j.metabol.2009.07.010 19766268 PMC2808028

[35] Warensjö E, Risérus U, Vessby B. Fatty acid composition of serum lipids predicts the development of the metabolic syndrome in men. Diabetologia. 2005; 48:1999–2005. 10.1007/s00125-005-1897-x 16132958

[36] Kotronen A, Velagapudi VR, Yetukuri L, Westerbacka J, Bergholm R, Ekroos K, Makkonen J, Taskinen MR, Oresic M, Yki-Järvinen H. Serum saturated fatty acids containing triacylglycerols are better markers of insulin resistance than total serum triacylglycerol concentrations. Diabetologia. 2009; 52:684–90. 10.1007/s00125-009-1282-2 19214471

[37] Novgorodtseva TP, Karaman YK, Zhukova NV, Lobanova EG, Antonyuk MV, Kantur TA. Composition of fatty acids in plasma and erythrocytes and eicosanoids level in patients with metabolic syndrome. Lipids Health Dis. 2011; 10:82. 10.1186/1476-511X-10-82 21595891 PMC3116500

[38] Börgeson E, McGillicuddy FC, Harford KA, Corrigan N, Higgins DF, Maderna P, Roche HM, Godson C. Lipoxin A4 attenuates adipose inflammation. FASEB J. 2012; 26:4287–94. 10.1096/fj.12-208249 22700871

[39] Su X, Feng X, Terrando N, Yan Y, Chawla A, Koch LG, Britton SL, Matthay MA, Maze M. Dysfunction of inflammation-resolving pathways is associated with exaggerated postoperative cognitive decline in a rat model of the metabolic syndrome. Mol Med. 2013; 18:1481–90. 10.2119/molmed.2012.00351 23296426 PMC3576477

[40] Yu D, Xu Z, Yin X, Zheng F, Lin X, Pan Q, Li H. Inverse Relationship between Serum Lipoxin A4 Level and the Risk of Metabolic Syndrome in a Middle-Aged Chinese Population. PLoS One. 2015; 10:e0142848. 10.1371/journal.pone.0142848 26565966 PMC4643896

[41] Pickens CA, Sordillo LM, Comstock SS, Harris WS, Hortos K, Kovan B, Fenton JI. Plasma phospholipids, non-esterified plasma polyunsaturated fatty acids and oxylipids are associated with BMI. Prostaglandins Leukot Essent Fatty Acids. 2015; 95:31–40. 10.1016/j.plefa.2014.12.001 25559239 PMC4361296

[42] Grant RW, Dixit VD. Adipose tissue as an immunological organ. Obesity (Silver Spring). 2015; 23:512–18. 10.1002/oby.21003 25612251 PMC4340740

[43] Careaga VP, Sacca PA, Mazza ON, Scorticati C, Vitagliano G, Fletcher SJ, Maier MS, Calvo JC. Fatty acid composition of human periprostatic adipose tissue from argentine patients and its relationship to prostate cancer and benign prostatic hyperplasia. Research in Cancer and Tumor. 2015; 4:1–6.

[44] Trifiro MD, Parsons JK, Palazzi-Churas K, Bergstrom J, Lakin C, Barrett-Connor E. Serum sex hormones and the 20-year risk of lower urinary tract symptoms in community-dwelling older men. BJU Int. 2010; 105:1554–59. 10.1111/j.1464-410X.2009.09090.x 20002438

[45] Parsons JK, Palazzi-Churas K, Bergstrom J, Barrett-Connor E. Prospective study of serum dihydrotestosterone and subsequent risk of benign prostatic hyperplasia in community dwelling men: the Rancho Bernardo Study. J Urol. 2010; 184:1040–44. 10.1016/j.juro.2010.05.033 20643424

[46] Grzesiak K, Rył A, Baranowska-Bosiacka I, Rotter I, Dołęgowska B, Słojewski M, Sipak-Szmigiel O, Ratajczak W, Lubkowska A, Metryka E, Piasecka M, Laszczyńska M. Comparison between selected hormone and protein levels in serum and prostate tissue homogenates in men with benign prostatic hyperplasia and metabolic disorders. Clin Interv Aging. 2018; 13:1375–82. 10.2147/CIA.S168146 30122909 PMC6080669

[47] Kaplan SA, O’Neill E, Lowe R, Hanson M, Meehan AG. Prevalence of low testosterone in aging men with benign prostatic hyperplasia: data from the Proscar Long-term Efficacy and Safety Study (PLESS). Aging Male. 2013; 16:48–51. 10.3109/13685538.2013.773421 23480623

[48] Bhasin S, Singh AB, Mac RP, Carter B, Lee MI, Cunningham GR. Managing the risks of prostate disease during testosterone replacement therapy in older men: recommendations for a standardized monitoring plan. J Androl. 2003; 24:299–311. 10.1002/j.1939-4640.2003.tb02676.x 12721204

[49] Parsons JK, Palazzi-Churas K, Bergstrom J, Barrett-Connor E. Prospective study of serum dihydrotestosterone and subsequent risk of benign prostatic hyperplasia in community dwelling men: the Rancho Bernardo Study. J Urol. 2010; 184:1040–44. 10.1016/j.juro.2010.05.033 20643424

[50] Prins GS, Birch L, Couse JF, Choi I, Katzenellenbogen B, Korach KS. Estrogen imprinting of the developing prostate gland is mediated through stromal estrogen receptor alpha: studies with alphaERKO and betaERKO mice. Cancer Res. 2001; 61:6089–97. 11507058

[51] Miwa Y, Kaneda T, Yokoyama O. Association between lower urinary tract symptoms and serum levels of sex hormones in men. Urology. 2008; 72:552–55. 10.1016/j.urology.2008.04.023 18597822

[52] Alberti KG, Zimmet P, Shaw J, and IDF Epidemiology Task Force Consensus Group. The metabolic syndrome--a new worldwide definition. Lancet. 2005; 366:1059–62. 10.1016/S0140-6736(05)67402-8 16182882

[53] Katsuki A, Sumida Y, Gabazza EC, Murashima S, Furuta M, Araki-Sasaki R, Hori Y, Yano Y, Adachi Y. Homeostasis model assessment is a reliable indicator of insulin resistance during follow-up of patients with type 2 diabetes. Diabetes Care. 2001; 24:362–65. 10.2337/diacare.24.2.362 11213893

[54] Folch J, Lees M, Sloane Stanley GH. A simple method for the isolation and purification of total lipides from animal tissues. J Biol Chem. 1957; 226:497–509. 13428781

